# Large Language Model–Based Simplification of Digital Therapeutics Explanations for Insomnia and Nicotine Dependence: Two Randomized Online Experiments

**DOI:** 10.2196/89451

**Published:** 2026-06-10

**Authors:** JunYoung Seo, Moses Yook, Dai Jin Kim, JunHee Lee, Jae Hyun Yoo, GiHwan Byeon, In Young Choi

**Affiliations:** 1 Department of Medical Informatics, College of Medicine, The Catholic University of Korea, 222, Banpo-daero, Seocho-gu, Seoul, Republic of Korea, 82 2-3147-8423; 2 Department of Psychiatry, College of Medicine, The Catholic University of Korea, Seoul, Republic of Korea

**Keywords:** digital therapeutics, large language models, text simplification, patient education materials, randomized online experiment

## Abstract

**Background:**

Digital therapeutics (DTx) are evidence-based software interventions with the potential to treat health conditions. However, uptake remains limited by low public awareness and overly complex patient education materials that exceed recommended readability levels. Large language models (LLMs) may simplify such content; however, their effects on users’ understanding have not been empirically demonstrated.

**Objective:**

This study aimed to examine whether LLM-based simplification of DTx explanatory materials enhances perceived understanding and subjective evaluations of readability, clarity, and comprehensibility compared with manufacturer-provided documents.

**Methods:**

We developed a simplification tool using the GPT-4o application programming interface (API), configured for deterministic outputs and guided by structured readability instructions. Original DTx explanatory materials about insomnia and nicotine dependence were obtained from manufacturers and transformed into simplified versions. Two randomized, between-subject online experiments were conducted (n=1000, with 500 participants in each experiment). Participants were stratified by age and sex and screened for relevance (Insomnia Severity Index ≥8 for the insomnia experiment and smoking ≥5 cigarettes per day for the nicotine dependence experiment). Within each experiment, participants were randomly assigned to review either the original or the LLM-simplified explanatory material. Perceived understanding was assessed before and after exposure. Postexposure evaluations of ease, clarity, and comprehensibility were also collected.

**Results:**

Repeated measures ANOVA revealed significant group×time interaction effects on perceived understanding in both experiments: insomnia (*F*_1,498_=24.8; *P*<.001) and nicotine dependence (*F*_1,498_=14.1; *P*<.001), with greater improvements observed in the LLM-simplified groups. Mann-Whitney *U* tests further showed that LLM-simplified explanations were rated as easier, clearer, and more comprehensible than the original versions in both experiments (all *P*<.05), with small to moderate effect sizes (*r*=0.11-0.24).

**Conclusions:**

Compared with manufacturer-provided original materials, LLM-simplified DTx explanations led to greater improvements in perceived understanding and subjective evaluations of readability among lay audiences, even after a single exposure. This finding highlights the potential scalability of LLM-based simplification as a strategy to improve the perceived accessibility of health information for lay audiences. Integrating such tools into patient education may enhance how lay audiences perceive and engage with DTx, although further research using objective comprehension measures is needed to confirm these benefits.

## Introduction

### Background

Digital therapeutics (DTx) represent a significant advancement in health care and are defined as high-quality software programs delivering evidence-based therapeutic interventions for a wide range of physical, mental, and behavioral conditions [1-3]. Unlike traditional pharmaceuticals and devices, DTx are often cost-effective and designed to encourage sustained user engagement, making them a promising avenue for modern health care [4,5]. DTx have been applied to various conditions, including chronic pain, cardiometabolic diseases (eg, diabetes and hypertension), heart failure, and mental health disorders. They demonstrate potential to complement pharmacotherapy; improve adherence; enable remote monitoring; and expand therapeutic options through emerging technologies, such as virtual reality and wearable sensors [6-10]. In this study, we focused on 2 DTx use cases, insomnia and nicotine dependence, as accessible and representative examples to evaluate the effects of large language model (LLM)–based simplification.

### Prior Work

Despite promising applications, the adoption of DTx remains limited [11,12]. Public awareness is low, and many individuals outside the medical community are unfamiliar with DTx or do not fully understand their purpose [13]. For new health technologies to achieve broader societal diffusion, individuals must develop a clear understanding of their value [14-16].

A significant barrier is the inherent complexity of medical information. Research indicates that the average health literacy level of US adults corresponds to that of 13- to 14-year-olds, and medical education materials are recommended to be written at the level of 10- to 11-year-olds [17]. However, over 95% of existing patient education materials require a reading level of at least high school, making them inaccessible to a significant portion of the general population [18]. Producing simplified yet accurate resources remains a resource-intensive and time-consuming process that requires substantial expertise [19-21].

LLMs offer a promising solution. Trained on vast amounts of text data, LLMs can perform natural language tasks, such as summarization, translation, and simplification [22]. Their ability to adapt content to different comprehension levels has been shown to improve the readability of health information, often quantified through automated metrics, such as Flesch-Kincaid grade levels [23-25]. However, these evaluations have rarely examined whether improved readability translates to greater comprehension or more positive user perceptions among lay audiences. This gap highlights the need for empirical studies that directly assess how individuals experience and interpret simplified LLM-generated health information.

### Study Objectives

This study aimed to address these gaps by empirically testing whether LLM-based simplification of DTx explanatory materials for insomnia and nicotine dependence enhances perceived understanding and subjective evaluations. Two outcome categories were assessed. The first was the cognitive outcome, which examined whether exposure to LLM-simplified explanations yields greater perceived understanding than exposure to the original explanations. The second was the subjective outcome, which examined whether LLM-based simplification improves evaluations of explanatory materials regarding ease, clarity, and overall comprehensibility.

Specifically, we aimed to determine whether LLM-simplified DTx explanations, compared with the original manufacturer-provided materials, lead to (1) greater improvements in perceived understanding and (2) higher subjective ratings of ease, clarity, and comprehensibility, across 2 independent randomized online experiments.

## Methods

### Study Design

This study used a randomized, between-subjects online experimental design to evaluate the effectiveness of LLM-simplified DTx explanations. Two independent experiments were conducted, each focusing on a different therapeutic area: insomnia or nicotine dependence.

In both experiments, participants were randomly assigned to one of two groups:

1. Control group—received the original manufacturer-provided DTx explanatory materials
2. Experimental group—received LLM-simplified versions of the same materials

This design allowed direct comparison of the effects of the original versus simplified explanations on participants’ perceived understanding and subjective evaluations.

### Development of the LLM-based Simplification Tool

To address the challenge of making DTx information more comprehensible to the public while maintaining clinical accuracy, we developed a lightweight text simplification tool. The tool used the GPT-4o (OpenAI) application programming interface (API), a state-of-the-art LLM, for its advanced natural language processing capabilities. The primary goal was to generate simplified text that would be understandable to lay audiences while ensuring fidelity to the original medical content.

In alignment with recommendations for health education materials that suggest a readability level suitable for 10- to 11-year-olds [17], the tool was specifically designed to simplify complex DTx explanations to this level, ensuring accessibility across diverse populations regardless of health literacy.

The simplification process was guided by structured instructions covering content preservation, logical coherence, terminology adaptation, clarity enhancement, tone and style, emphasis, length management, and readability. The full prompt text provided to the LLM is available in Multimedia Appendix 1.

For model configuration, the temperature parameter was set to 0 to minimize randomness and maximize reproducibility, and the top p parameter was fixed at 1 to ensure comprehensive consideration of relevant tokens. This deterministic setup was critical for maintaining precision and consistency in the medical information. These design choices enabled the creation of a tool that converts complex DTx documents into simplified, patient-friendly explanations without compromising medical accuracy.

### DTx Explanatory Materials

To evaluate the generalizability of the proposed text simplification approach, we selected 2 DTx that addressed distinct health conditions: insomnia and nicotine dependence. This design enabled the assessment of the applicability of the LLM-based simplification tool across different therapeutic domains, rather than within a single disease context.

For each DTx, original patient education and explanatory documents were obtained directly from the respective manufacturers. These materials, characterized by high complexity and technical terminology, served as baseline texts for the simplification process. Using the LLM-based tool described above, simplified versions of each document were generated, resulting in two parallel sets of materials for each DTx product: (1) the original manufacturer-provided version and (2) the LLM-simplified version.

The LLM-simplified versions incorporated several modifications, including vocabulary simplification, tone adjustment, and formatting improvements, such as clearer paragraph structuring and emphasis of key points. In contrast, the original manufacturer-provided materials were presented in their original plain-text format without any modification.

To ensure medical accuracy and logical coherence, all LLM-generated materials were independently reviewed by a panel of 3 board-certified psychiatrists who regularly treat conditions such as insomnia and nicotine dependence. This expert validation confirmed that the simplified content faithfully preserved the original medical meaning, avoided factual errors or misinterpretations, and presented information in a logically coherent manner. This review process was essential to maintaining the clinical integrity and trustworthiness of the materials used in this study. The full texts of the original and LLM-simplified explanatory materials are provided in Multimedia Appendix 2.

### Participants and Recruitment

In total, 1000 participants were recruited through an online panel service (Macromill Embrain Co, Ltd) [26], with 500 participants allocated to each experiment (n=500, 50% to insomnia DTx and n=500, 50% to nicotine dependence DTx). Stratified assignment was applied during recruitment to ensure comparable sex and age distributions between the control and experimental groups.

The general inclusion criteria were (1) age between 19 and 64 years, (2) access to a smartphone or computer with internet connectivity, and (3) provision of informed consent before participation. Condition-specific screening thresholds were established in consultation with 3 board-certified psychiatrists to enhance contextual relevance while recruiting participants from the general population rather than from patient groups. For the insomnia DTx experiment, only participants with an Insomnia Severity Index score of ≥8 were included, corresponding to at least mild insomnia symptoms [27]. The Insomnia Severity Index is a 7-item self-report scale assessing insomnia symptom severity, with total scores ranging from 0 to 28. For the nicotine dependence DTx experiment, only participants who reported smoking at least 5 cigarettes per day were eligible. These criteria were selected to ensure that participants, although not recruited as patients, shared health-related characteristics that made the DTx materials meaningful and relevant to their personal contexts.

### Procedure

This study used an online, randomized, between-subjects experimental design. Participants were recruited through an online panel service and screened according to the general and condition-specific inclusion criteria described above. Only individuals who met eligibility requirements were invited to participate.

After confirming eligibility, participants provided digital informed consent. In each experiment, participants were recruited from an online panel service [26] that collected demographic information (age and sex) during the screening process. To ensure balance across conditions, stratified randomization was applied, assigning participants to either the control group (original materials) or the experimental group (LLM-simplified materials) within each demographic stratum. This approach minimized allocation bias and maintained between-group comparability.

Following the group assignment, participants completed a structured online questionnaire in the following sequence:

- Pre-exposure assessment of perceived understanding of the respective DTx
- Exposure to the assigned explanatory materials (original vs LLM-simplified) with a minimum enforced viewing time of 30 seconds
- Postexposure assessment of perceived understanding of the respective DTx
- Evaluation of explanations, where participants rated the difficulty, clarity, and overall comprehensibility of the materials

All responses were anonymized to ensure the strict protection of participant privacy. After completing the survey, participants received compensation according to the agreement established by the online panel service.

### Measures

#### Perceived Understanding

Participants’ perceived understanding of the 2 DTx was assessed both before and after exposure to the explanatory materials. This construct was measured using 5 items adapted from Flynn and Goldsmith subjective knowledge scale [28], modified to capture how much participants believed they understood each DTx. Responses were recorded on a 5-point Likert scale ranging from 0 (not at all) to 4 (very much). The exact wording of the 5 adapted items is provided in the Multimedia Appendix 3. The scale demonstrated good internal consistency, with Cronbach α=0.80 for the insomnia DTx experiment and α=0.82 for the nicotine dependence DTx experiment.

#### Evaluation of Explanations

Participants’ evaluation of the explanatory materials was measured after exposure using single-item ratings on a 0 to 4 scale, where 0 indicated “not at all” and 4 indicated “very much.” Three aspects were assessed: (1) ease (how easy or difficult the explanation was to read and follow), (2) clarity (how clear the explanation was perceived overall), and (3) comprehensibility (the extent to which participants felt they could understand the explanation).

All questionnaires were administered in Korean. No objective knowledge tests (eg, recall or factual questions) were included; thus, all outcome measures relied on participants’ subjective self-reports.

### Ethical Considerations

This study was approved by the Institutional Review Board of the Catholic Medical Center (approval MC25QNSI0039) and was conducted in accordance with the principles of the Declaration of Helsinki [29]. All participants provided informed consent before participation. All participant data were anonymized and kept strictly confidential; no personally identifiable information was collected or retained. Participants received compensation for their participation in accordance with the terms established by the online panel service [26]. This study is reported in accordance with the Consolidated Standards of Reporting Trials of Electronic and Mobile Health Applications and Online Telehealth (CONSORT-EHEALTH) guidelines; the completed checklist is provided in Checklist 1.

### Statistical Analysis

All statistical analyses were conducted using Jamovi (version 2.6.26). A significance threshold of α=.05 was applied to all tests. To examine the effect of explanation type on perceived DTx understanding, repeated measures ANOVA was conducted. Each model tested the group (LLM-simplified vs control)×time (before vs after exposure) interaction to determine whether changes in perceived understanding over time significantly differed between groups. The assumptions of normality, homoscedasticity, and sphericity were satisfied.

Participants’ postexposure ratings of ease, clarity, and comprehensibility of the DTx explanations were compared between groups using the Mann-Whitney *U* test. This nonparametric approach was selected because of the ordinal nature of the single-item Likert-scale ratings. These analyses evaluated whether LLM-based simplification had a significant influence on participants’ subjective evaluations of the materials.

## Results

### Sample Characteristics

A total of 1000 participants completed the study, with 500 (50%) allocated to the insomnia DTx experiment and 500 (50%) to the nicotine dependence DTx experiment. Stratified assignment ensured comparable sex and age distributions between the control and experimental groups. Demographic information used for stratification was limited to sex and age. Participant demographic characteristics are presented in Table 1.

**Table 1.** Participant demographic characteristics by group (insomnia and nicotine dependence experiments).

Characteristic	Insomnia control (n=250)	Insomnia test (n=250)	Nicotine control (n=250)	Nicotine test (n=250)
Sex, n (%)				
Male	125 (50)	125 (50)	125 (50)	125 (50)
Female	125 (50)	125 (50)	125 (50)	125 (50)
Age (years), n (%)				
20	63 (25.2)	63 (25.2)	63 (25.2)	63 (25.2)
30	63 (25.2)	63 (25.2)	63 (25.2)	63 (25.2)
40	62 (24.8)	62 (24.8)	62 (24.8)	62 (24.8)
50-65	62 (24.8)	62 (24.8)	62 (24.8)	62 (24.8)

### Effect on Perceived Understanding

No significant main effect of group was observed in either the insomnia experiment (*F*_1,498_=3.32; *P*=.07; partial η^2^=0.007) or the nicotine dependence experiment (*F*_1,498_=2.88; *P*=.09; partial η^2^=0.006; Table 2).

**Table 2.** Repeated measures ANOVA results for perceived understanding across insomnia and nicotine dependence digital therapeutics (DTx) experiments.

Experiment and effect	*F* test (*df*)	*P* value	Partial η^2^
Insomnia DTx			
Group	3.32 (1, 498)	.07	0.007
Time	38.5 (1, 498)	<.001	0.072
Group×time	24.8 (1, 498)	<.001	0.048
Nicotine DTx			
Group	2.88 (1, 498)	.09	0.006
Time	21.5 (1, 498)	<.001	0.041
Group×time	14.1 (1, 498)	<.001	0.028

A significant main effect of time was observed in both the insomnia experiment (*F*_1,498_=38.5; *P*<.001; partial η^2^=0.072) and the nicotine dependence experiment (*F*_1,498_=21.5, *P*<.001; partial η^2^=0.041), with postexposure assessment scores higher than pre-exposure assessment scores across groups.

A significant group×time interaction was observed in both experiments. In the insomnia experiment, the interaction effect was *F*_1, 498_=24.8; *P*<.001; partial η^2^=0.048; and in the nicotine dependence experiment, it was *F*_1, 498_=14.1; *P*<.001; partial η^2^=0.028 (Table 2).

**Figure 1.**
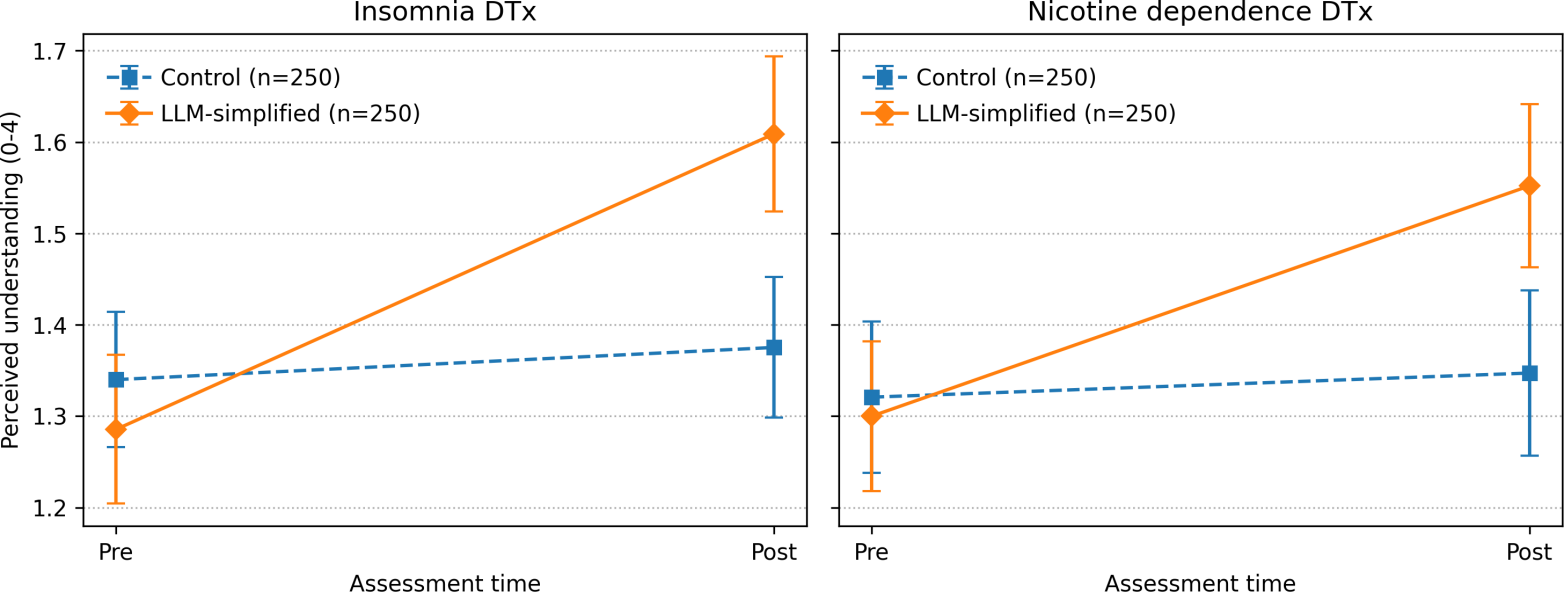
Changes in perceived digital therapeutics (DTx) understanding from pre-exposure assessment to postexposure assessment in both experiments. Points indicate group means, and error bars represent 95% CIs. LLM: large language model.

Descriptive statistics for each group and time point are presented in Table 3. As shown in Figure 1, the LLM-simplified group demonstrated greater increases in perceived understanding than the control group in both experiments.

**Table 3.** Perceived understanding scores by group and time point (n=250).

Experiment and group	Pretest, mean (SD; 95% CI)	Posttest, mean (SD; 95% CI)
Insomnia DTx^a^		
LLM^b^-simplified	1.29 (0.65; 1.20-1.37)	1.61 (0.68; 1.52-1.69)
Control	1.34 (0.59; 1.27-1.41)	1.38 (0.62; 1.30-1.45)
Nicotine DTx		
LLM-simplified	1.30 (0.66; 1.22-1.38)	1.55 (0.72; 1.46-1.64)
Control	1.32 (0.66; 1.24-1.40)	1.35 (0.73; 1.26-1.44)

a DTx: digital therapeutics.

b LLM: large language model.

### Evaluation of Explanations

In the insomnia experiment, the LLM-simplified explanations were rated significantly higher for ease (*U*=27,281; *P*=.008; *r*=0.127), clarity (*U*=27,803; *P*=.02; *r*=0.110), and comprehensibility (*U*=25,125; *P*<.001; *r*=0.196).

**Figure 2.**
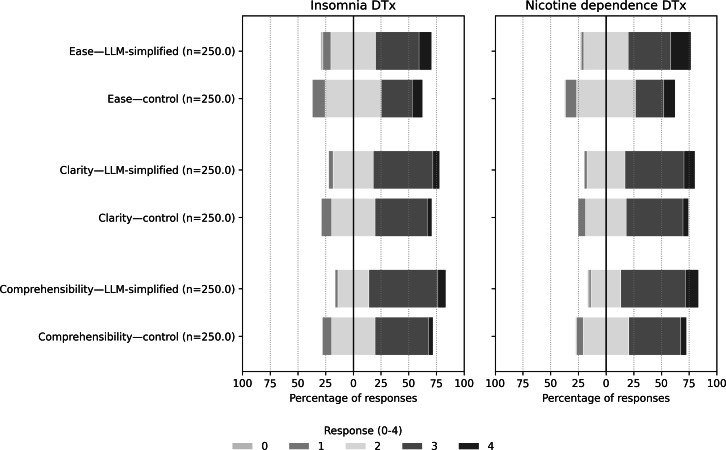
Group distribution of postexposure ratings (0‐4) for ease, clarity, and comprehensibility in both experiments. Bars represent 100% stacked proportions. The neutral category (2) is split and plotted on both sides of the center, such that lower ratings (0‐1) extend to the left of 0 and higher ratings (3-4) extend to the right. This split-neutral presentation allows the direction of responses (negative vs positive) to be visually compared while preserving the full distribution of ratings. Group labels indicate sample sizes (control vs large language model [LLM]–simplified). Between-group differences were tested using 2-sided Mann-Whitney *U* tests with Holm correction across measures; rank-biserial effect sizes (*r*) are reported in Table 4. DTx: digital therapeutics.

Similarly, in the nicotine dependence experiment, significant group differences were observed for ease (*U*=23,727; *P*<.001; *r*=0.240), clarity (*U*=27,697; *P*=.02; *r*=0.114), and comprehensibility (U=24,738; *P*<.001; *r*=0.208). The distribution of ratings across response categories is illustrated in Figure 2.

**Table 4.** Mann-Whitney *U* test results comparing subjective evaluation of explanations between groups.

Experiment and measure	*U*	*P* value	Adjusted *P* value^a^	*r*
Insomnia DTx^b^				
Ease	27,281	.008	.02	0.127
Clarity	27,803	.02	.03	0.11
Comprehensibility	25,125	<.001	.006	0.196
Nicotine DTx				
Ease	23,727	<.001	.006	0.24
Clarity	27,697	.02	.03	0.114
Comprehensibility	24,738	<.001	.006	0.208

a Holm correction applied for multiple comparisons.

b DTx: digital therapeutics.

## Discussion

This study evaluated whether LLM-based simplification of DTx explanatory materials improves perceived understanding and subjective evaluations among lay audiences through 2 independent randomized online experiments targeting insomnia (500/1000, 50%) and nicotine dependence (500/1000, 50%).

### Principal Results

Our findings indicated that LLM-based simplification of DTx explanatory materials improves perceived comprehensibility and subjective evaluations of accessibility. In 2 independent experiments on insomnia and nicotine dependence, participants who received LLM-simplified explanations reported significantly greater gains in perceived understanding than those who received the original manufacturer-provided explanations. In addition, the simplified versions were consistently rated as easier, clearer, and more comprehensible, indicating improved subjective evaluations. These findings suggest that LLM-based simplification may improve perceived understanding and subjective evaluations across multiple therapeutic contexts. However, further research using objective comprehension measures and more diverse populations is needed to confirm the robustness and generalizability of these findings.

Although these findings demonstrate improvements in perceived understanding, these improvements do not necessarily indicate accurate or complete comprehension of medical information. Feeling confident about understanding health information may differ from actually understanding the details and appropriate use of a medical intervention. Therefore, future research should incorporate objective knowledge measures to determine whether LLM-based simplification improves actual comprehension in addition to perceived understanding.

The observed differences in subjective evaluations were statistically significant, but the effect sizes were generally small to moderate (*r*=0.11‐0.24). These magnitudes suggest modest but consistent improvements in participants’ evaluations following a single brief exposure. Given the minimal intervention required, such improvements may still be meaningful in real-world settings where scalable approaches to improving the perceived accessibility of health information are needed.

These findings emphasize the potential for safe and practical applications of LLMs in health care communication. Although concerns about hallucinations highlight the risks of using LLMs to generate new clinical content or make medical decisions [30], their role in transforming clinically validated information into more accessible formats carries considerably lower risk [31]. Prior research has demonstrated that readability can be improved while maintaining factual accuracy [23-25]. This study extends that evidence by incorporating independent reviews from 3 board-certified psychiatrists who confirmed the medical accuracy and logical coherence of the simplified texts. Collectively, these results demonstrate that LLM-based simplification represents a scalable and trustworthy strategy for improving the perceived accessibility and comprehensibility of DTx information among lay audiences.

### Comparison With Prior Work

The findings of this study align with a substantial body of research that highlights the challenges of health literacy and the frequent mismatch between the readability of medical information and patients’ comprehension skills [17]. Previous studies have consistently shown that most patient education materials are written at a level above the recommended reading level, limiting accessibility for the general population [18]. Recent advances in natural language processing and LLMs suggest that automated text simplification can serve as a promising solution, with evidence demonstrating improvements in readability across a range of health-related documents [22]. However, much of this prior work relied on computational readability metrics (such as Flesch-Kincaid scores) rather than empirical evaluations with end users [23-25].

This study extends this line of research by providing experimental evidence that LLM-simplified explanations not only achieve higher readability but also enhance perceived understanding among lay audiences. Notably, the results demonstrate that accuracy can be preserved, consistent with prior findings that LLM-based simplification improves accessibility without compromising factual correctness [23-25]. Unlike earlier studies, this study combines expert clinical validation by board-certified psychiatrists with large-scale user experiments across 2 therapeutic contexts, thereby offering novel empirical evidence of both accuracy and effectiveness. These findings reinforce the growing recognition that LLM-based simplification may help improve the perceived accessibility of DTx information and support users’ perceived understanding of complex health technologies.

### Limitations

This study has some limitations. First, participants were recruited from an online panel and allocated through stratified randomization based on sex and age, which may have reduced natural variability and limited the generalizability of the results [32]. Because participation required internet access and familiarity with digital environments, the study sample may have had higher digital literacy than typical patient populations. Therefore, the findings may not fully generalize to individuals with limited digital literacy. In addition, because demographic information was limited to sex and age, other potentially relevant characteristics (such as education, income, health literacy, and prior DTx exposure) were not collected, further limiting the interpretability of the results. In particular, differences in education level and baseline health literacy may influence how individuals respond to simplified health information and should be considered when interpreting the generalizability of the findings. Second, comprehension was assessed only through self-reported perceived understanding rather than objective knowledge tests [33], which limits conclusions about actual comprehension. Third, ease, clarity, and comprehensibility were evaluated using single-item measures, restricting measurement reliability. Fourth, the short-term exposure with a minimum enforced viewing time of 30 seconds leaves the durability and behavioral relevance of the effects uncertain. Fifth, although participants were screened for relevance (eg, minimum insomnia symptoms or daily smoking frequency), they were not clinically diagnosed; thus, caution is warranted when extrapolating these findings to treatment populations. Sixth, the intervention relied on a single LLM (GPT-4o) and specific prompt configurations, underscoring the need for replication using alternative models and implementation conditions to establish robustness. Finally, the intervention involved multiple simultaneous modifications, including vocabulary simplification, tone adjustment, and formatting improvements, such as paragraph structuring and emphasis of key points. Notably, the original manufacturer-provided materials were presented in plain text without visual enhancements, whereas the LLM-simplified versions incorporated formatting elements, such as bolding, structured paragraph breaks, and emphasis of key terms, as part of the simplification process. Therefore, the comparison was not purely linguistic; differences in visual presentation may have independently contributed to the observed improvements in perceived comprehensibility and subjective evaluations. This constitutes a bundled intervention, and the present design does not allow for disentangling the effects of linguistic simplification from those of visual formatting. Future studies should isolate these components—for example, through factorial designs that independently vary linguistic complexity and visual formatting—to better understand their individual and combined effects.

### Conclusions

This study demonstrates that LLM-based simplification of DTx explanatory materials significantly improves perceived understanding and subjective evaluations of readability, clarity, and comprehensibility among lay audiences. By focusing on the transformation of clinically validated content, LLMs can provide a safe and scalable approach to improving the perceived accessibility of digital health information.

Beyond DTx, these findings have broader implications: many types of medical documents, including patient education materials, informed consent forms, and clinical instructions, can be made to feel more accessible through LLM-based simplification. Importantly, by helping patients feel more confident in engaging with health information, LLM-based tools may have the potential to reduce passive compliance and instead promote active engagement, adherence, and shared decision-making.

Future studies should extend these results in several ways. Research involving clinically diagnosed patient populations is necessary to confirm the applicability of this approach in real-world treatment settings. Additionally, objective knowledge assessments and longitudinal designs are needed to determine whether improvements in perceived understanding translate into sustained learning and behavioral change. Finally, evaluations across diverse health conditions, populations, and languages are essential to establish the robustness and generalizability of LLM-based simplification strategies.

## Supplementary material

10.2196/89451 Multimedia Appendix 1 Full prompt text used for the large language model.

10.2196/89451 Multimedia Appendix 2 Original and large language model–simplified explanatory materials.

10.2196/89451 Multimedia Appendix 3 Survey questionnaire.

10.2196/89451 Checklist 1 CONSORT-EHEALTH (V 1.6.1) checklist for reporting randomized trials of eHealth interventions.

## Abbreviations

API: application programming interface
CONSORT-EHEALTH: Consolidated Standards of Reporting Trials of Electronic and Mobile Health Applications and Online Telehealth
DTx: digital therapeutics
LLM: large language model
