# Supplementary material for: Large Language Model–Based Simplification of Digital Therapeutics Explanations for Insomnia and Nicotine Dependence: Two Randomized Online Experiments
Source: JMIR Hum Factors. 2026 Jun 10;13:e89451. doi: 10.2196/89451 (PMC13252706; doi:10.2196/89451)
Supplement: Multimedia Appendix 1 [file humanfactors-v13-e89451-s001.docx]

**Appendix 1**. Full prompt text used for the large language model

Please rewrite the following text so that it can be easily understood by a 10-year-old child.

Follow these instructions carefully:

- Do not omit any information. All details and meanings from the original text must be included.
- Keep the logical flow of the text, including cause-and-effect relationships and conditions.
- Replace technical or medical jargon with simpler words, but do not weaken or distort the original medical meaning or core concepts.
- If a part could be confusing, add a short example or analogy to make it clearer.
- Use a tone that is kind and calm, like a doctor explaining to a patient’s family member.
- Highlight important points in bold.
- The length should be close to the original text, not excessively longer.
- Use paragraph breaks, spacing, and line separation to improve readability.
- Avoid vague expressions. If necessary, add clarifying remarks such as, “This means that …” to ensure accuracy.
