# Supplementary material for: Large Language Model–Based Simplification of Digital Therapeutics Explanations for Insomnia and Nicotine Dependence: Two Randomized Online Experiments
Source: JMIR Hum Factors. 2026 Jun 10;13:e89451. doi: 10.2196/89451 (PMC13252706; doi:10.2196/89451)
Supplement: Multimedia Appendix 2 [file humanfactors-v13-e89451-s002.docx]

**Appendix 2.** Original and LLM-simplified explanatory materials

# Original Explanatory Material (Insomnia DTx)

| This product is a software -based medical device designed for the treatment of chronic insomnia. It delivers the cognitive behavioral therapy for insomnia (CBT-I) protocol, the gold-standard treatment in clinical practice through a mobile application that applies a structured algorithm sequentially. The CBT-I protocol includes stimulus control, sleep restriction, sleep hygiene education, relaxation training, and cognitive therapy.  Cognitive behavioral therapy is a psychotherapeutic approach that alleviates symptoms by modifying maladaptive thoughts and behaviors. The cognitive component targets dysfunctional beliefs related to insomnia, thereby promoting healthy sleep habits, whereas the behavioral component, through techniques such as stimulus control, sleep restriction, and relaxation training aims to improve sleep quality.  Based on these principles, the application provides patients with structured education, real-time feedback, behavioral interventions, and push notifications over a 6- to 9-week period. These interventions are intended to increase sleep efficiency and, ultimately, alleviate symptoms of insomnia. |
| --- |

# LLM-Simplified Explanatory Material (Insomnia DTx)

| This product is special software designed to help people who have trouble sleeping. It uses a method called **cognitive behavioral therapy for insomnia** (CBT-I). This method is widely used in hospitals and clinics to treat people who cannot sleep well.  The software applies several techniques. For example, **stimulus control** guides you on what to do and what to avoid before bedtime. **Sleep restriction** helps adjust your sleep schedule so you can rest more deeply. **Sleep hygiene education** teaches healthy bedtime habits**. Relaxation training** helps your body and mind feel calm. Finally, **cognitive therapy** helps change unhelpful thoughts about sleep.  You can use this therapy through a smartphone app. For 6 to 9 weeks, the app provides sleep lessons, gives real-time feedback, and supports behavior change. It also sends reminders so you do not forget to practice. In this way, the program helps you sleep better and, over time, reduce symptoms of insomnia.  Simply put, this software is like a personal sleep coach. It supports you day by day to help you build better sleep habits. |
| --- |

# Original Explanatory Material (Nicotine Dependence DTx)

| This product is a digital therapeutic (DTx) software designed for the treatment of nicotine dependence, aiming to reduce symptoms of nicotine addiction. It is delivered as a mobile medical application over an 8-week period and applies evidence-based clinical protocols of motivational enhancement therapy (MET) and cognitive behavioral therapy (CBT) using artificial intelligence (AI) chatbot and algorithmic technologies. The app was developed to expand treatment accessibility for individuals with nicotine use disorder outside hospital outpatient settings, while also improving workflow efficiency for healthcare providers. In this way, it offers an innovative and cost-effective therapeutic intervention. This product is prescribed for patients clinically diagnosed with nicotine use disorder, including those with mental and behavioral conditions caused by cigarette smoking. Its core functionality is based on MET and CBT principles, delivered through AI chatbot and algorithmic technologies embedded within the mobile application. The AI chatbot engages patients in interactive conversations to build rapport and encourage adherence. By continuously monitoring smoking frequency, craving intensity, and medication adherence, the system predicts relapse risk and enables timely interventions. Ultimately, the application aims to lower relapse rates, extend treatment duration, and support long-term smoking cessation. |
| --- |

# LLM-Simplified Explanatory Material (Nicotine Dependence DTx)

| This **digital therapeutic** app is designed to help people who want to quit smoking. It is a special kind of program that supports individuals with **nicotine dependence** so they can gradually reduce smoking and, eventually, stop completely.  The program lasts for **8 weeks** and uses two main approaches. The first is called **motivational enhancement therapy**, which strengthens your motivation and willpower to quit. The second is **cognitive behavioral therapy**, which teaches practical strategies for handling cravings and knowing what to do when you feel the urge to smoke.  The app also includes an **AI chatbot**, a smart digital companion you can talk to. For example, if you say, “I really want to smoke right now,” the chatbot might respond, “When you feel that way, try doing this instead.”  Because you can use this app at home, you do not need to visit the hospital or clinic every time. This **saves time and money** while also helping doctors provide support to more people.  The app also tracks how much you smoke, how strong your cravings are, and whether you are taking your medication. By monitoring these things, it can notice when you may be at risk of relapse and provide timely support to help you stay on track. In this way, it helps you extend your smoke-free time and makes it easier to **continue your quit attempt**.  This app is a prescription **digital therapeutic**, meaning you can use it when your doctor prescribes it. If you are trying to quit smoking, it may be helpful to discuss with your doctor whether this app could support your quit plan. |
| --- |
