# Supplementary material for: Large Language Model–Based Simplification of Digital Therapeutics Explanations for Insomnia and Nicotine Dependence: Two Randomized Online Experiments
Source: JMIR Hum Factors. 2026 Jun 10;13:e89451. doi: 10.2196/89451 (PMC13252706; doi:10.2196/89451)
Supplement: Multimedia Appendix 3 [file humanfactors-v13-e89451-s003.docx]

**Appendix 3.** Survey questionnaire

Screening Questionnaire

# Age

Q1. What is your current age (in full years)?

Eligible: Participants aged 19–64 years

# Screening for Insomnia (Insomnia Severity Index, ISI)

Over the last two weeks, please rate the **severity** of your sleep problems.

Q2-1. Difficulty falling asleep

0 = None

1 = Mild

2 = Moderate

3 = Severe

4 = Very severe

Q2-2. Difficulty staying asleep

0 = None

1 = Mild

2 = Moderate

3 = Severe

4 = Very severe

Q2-3. Problem waking up too early

0 = None

1 = Mild

2 = Moderate

3 = Severe

4 = Very severe

Q2-4. Satisfaction with current sleep pattern

0 = Very satisfied

1 = Satisfied

2 = Moderately satisfied

3 = Dissatisfied

4 = Very dissatisfied

Q2-5. Interference of sleep problems with daily functioning (eg, fatigue, mood, concentration, work performance, etc.)

0 = Not at all interfering

1 = A little interfering

2 = Somewhat interfering

3 = Much interfering

4 = Very much interfering

Q2-6. Noticeability of sleep problems to others

0 = Not at all noticeable

1 = A little noticeable

2 = Somewhat noticeable

3 = Much noticeable

4 = Very much noticeable

Q2-7. Degree of worry or distress caused by sleep problems

0 = Not at all worried

1 = A little worried

2 = Somewhat worried

3 = Much worried

4 = Very much worried

Scoring rule: Participants scoring ≥8 (total ISI score) are eligible for the insomnia DTx survey.

# Screening for Nicotine Dependence

Q3-1. Do you currently smoke cigarettes?

☐ Yes

☐ No

(If “No,” skip the following question.)

Q3-2. On average, how many cigarettes do you smoke per day during the past year?

☐ Fewer than 5 per day

☐ 5 or more per day

**Eligibility rule:** Participants who reported smoking **≥5 cigarettes/day** in the past year are eligible for the nicotine dependence DTx survey.

Survey on Digital Therapeutics for Insomnia

# 1. Pre-survey (Before exposure to information)

## 1-1. Demographics

Q1-1-1. What is your age group?

☐ 20s

☐ 30s

☐ 40s

☐ 50s

☐ 60s

Q1-1-2. What is your gender?

☐ Male

☐ Female

## 1-2. Perceived Understanding of Insomnia DTx (0 = Strongly disagree, 4 = Strongly agree)

The following questions ask about your knowledge and perceptions regarding digital therapeutics (DTx) for insomnia.

Please answer honestly based on your current impressions, even if you have never used such a device or been directly exposed to related information.

Q1-2-1. I think I know quite a lot about digital therapeutics for insomnia.

Q1-2-2. I do not think I have sufficient knowledge about digital therapeutics for insomnia. (reverse coded)

Q1-2-3. Compared with others, I believe I know more about digital therapeutics for insomnia.

Q1-2-4. I think I lack knowledge about digital therapeutics for insomnia. *(reverse coded)*

Q1-2-5. I do not know much about digital therapeutics for insomnia. *(reverse coded)*

# 2. Stimulus Presentation

Before completing the post-exposure questionnaire, participants were randomly assigned to one of two groups:

Group A: Received the original explanatory material

Group B: Received the LLM-simplified explanatory material

The following section contains explanatory material about a digital therapeutic for insomnia. Please read the text carefully.

# 3. Post-survey (After exposure to the explanatory material)

The following questions ask about your current perceptions **after reading the explanatory material**. Please respond based on how you feel at this moment.

## 3-1. Perceived Understanding of Insomnia DTx

Q3-1-1. I think I know quite a lot about digital therapeutics for insomnia.

Q3-1-2. I do not think I have sufficient knowledge about digital therapeutics for insomnia. (reverse coded)

Q3-1-3. Compared with others, I believe I know more about digital therapeutics for insomnia.

Q3-1-4. I think I lack knowledge about digital therapeutics for insomnia. *(reverse coded)*

Q3-1-5. I do not know much about digital therapeutics for insomnia. *(reverse coded)*

## 3-2. Evaluation of the Explanatory Material

Q1. How difficult was the explanatory material you just read? *(reverse coded)*
*(0 = Very easy, 4 = Very difficult)*

Q2. How clear did you find the explanatory material overall?
*(0 = Not at all clear, 4 = Very clear)*

Q3. How understandable was the explanatory material to you?
*(0 = Not at all understandable, 4 = Very understandable)*

Survey on Digital Therapeutics for Nicotine Dependence

# 1. Pre-survey (Before exposure to information)

## 1-1. Demographics

Q1-1-1. What is your age group?

☐ 20s

☐ 30s

☐ 40s

☐ 50s

☐ 60s

Q1-1-2. What is your gender?

☐ Male

☐ Female

## 1-2. Perceived Understanding of Nicotine Dependence DTx (0 = Strongly disagree, 4 = Strongly agree)

The following questions ask about your knowledge and perceptions regarding digital therapeutics (DTx) for nicotine dependence.

Please answer honestly based on your current impressions, even if you have never used such a device or been directly exposed to related information.

Q1-2-1. I think I know quite a lot about digital therapeutics for nicotine dependence.

Q1-2-2. I do not think I have sufficient knowledge about digital therapeutics for nicotine dependence. (reverse coded)

Q1-2-3. Compared with others, I believe I know more about digital therapeutics for nicotine dependence.

Q1-2-4. I think I lack knowledge about digital therapeutics for nicotine dependence. *(reverse coded)*

Q1-2-5. I do not know much about digital therapeutics for nicotine dependence. *(reverse coded)*

# 2. Stimulus Presentation

Before completing the post-exposure questionnaire, participants were randomly assigned to one of two groups:

Group A: Received the original explanatory material

Group B: Received the LLM-simplified explanatory material

The following section contains explanatory material about a digital therapeutic for nicotine dependence. Please read the text carefully.

# 3. Post-survey (After exposure to the explanatory material)

The following questions ask about your current perceptions **after reading the explanatory material**. Please respond based on how you feel at this moment.

## 3-1. Perceived Understanding of Nicotine Dependence DTx

Q3-1-1. I think I know quite a lot about digital therapeutics for nicotine dependence.

Q3-1-2. I do not think I have sufficient knowledge about digital therapeutics for nicotine dependence. (reverse coded)

Q3-1-3. Compared with others, I believe I know more about digital therapeutics for nicotine dependence.

Q3-1-4. I think I lack knowledge about digital therapeutics for nicotine dependence. *(reverse coded)*

Q3-1-5. I do not know much about digital therapeutics for nicotine dependence. *(reverse coded)*

## 3-2. Evaluation of the Explanatory Material

Q1. How difficult was the explanatory material you just read? *(reverse coded)*
*(0 = Very easy, 4 = Very difficult)*

Q2. How clear did you find the explanatory material overall?
*(0 = Not at all clear, 4 = Very clear)*

Q3. How understandable was the explanatory material to you?
*(0 = Not at all understandable, 4 = Very understandable)*
